# Suicide rate variation in Río Salaquí indigenous reservation among 1999 y 2019: a poblacional study

**DOI:** 10.1192/j.eurpsy.2023.1060

**Published:** 2023-07-19

**Authors:** S. Ghiso Jiménez, A. J. Rojas Sarmiento

**Affiliations:** 1 Psiquiatría, FUNIPAS, Medellín; 2 Enfermería, Blue Care Medplus, Bogotá, Colombia

## Abstract

**Introduction:**

Suicide, was defined as the act of intentionally taking one’s own life, understanding the lethal consequences of the act committed. The global suicide rate is 10.5 cases per 100,000 inhabitants, being lower in America, where it reaches values of 7.3 deaths per 100,000 inhabitants.

The Embera ethnic group is a Latin American indigenous people, of Chocó ethnolinguistic origin, which is located in the countries of Panama, Colombia and Ecuador; Particularly within the Embera ethnic group, a phenomenon of suicide in waves has been documented in journalistic media, which has been called “The Epidemic of the ropes”.

The data was collected in 2020, within the Río Salaquí-Isletas indigenous reservation (Riosucio-Chocó), in which multiple cases of suicide have occurred in the new millennium, without knowledge of previous cases.

**Objectives:**

To calculate the suicide rate and its trend over time in the Río Salaquí indigenous reservation, among 1999 and 2019 and compare the suicide rate and its trend with official data from Colombia, determining differences between the two.

**Methods:**

A descriptive study with an analytical component will be carried out. The data was collected in 2020, within the Río Salaquí-Isletas indigenous reservation (Riosucio-Chocó-Colombia). The information was taked from no structurated interviews conducted with individuals from the community.

The validation of the cases and the selection of duplicate cases was carried out through checklists created by the researchers. When calculating the specific suicide rates, the estimated population in the community is obtained from official censuses and local authorities.

Statistical analysis to determine whether the suicide rate; It was carried out with the SPSS program, using a confidence interval on the rate, which would allow estimating its variability with 95% reliability.

**Results:**

Data of deaths by suicide were collected in people of any age, residents of the Río Salaquí Indigenous Reservation (Isletas), between the decade of 1999 and 2019, as well as the total population of the community for the calculation of suicide rates. suicide.

Among the 10 years evaluated, 22 deaths by suicide were found, of which 13 (59%) corresponded to men and 9 (41%) to women. The suicide rate in Río Salaquí for this decade was 88.8 cases per 100,000 inhabitants 95% CI (68.7-375.8), with peaks in some three-year periods analyzed and no deaths by suicide before 2001. On the other hand, the The national suicide rate in Colombia for this decade was 4.56 cases per 100,000 inhabitants 95% CI (4.18-4.87).

**Image:**

**Figure:**
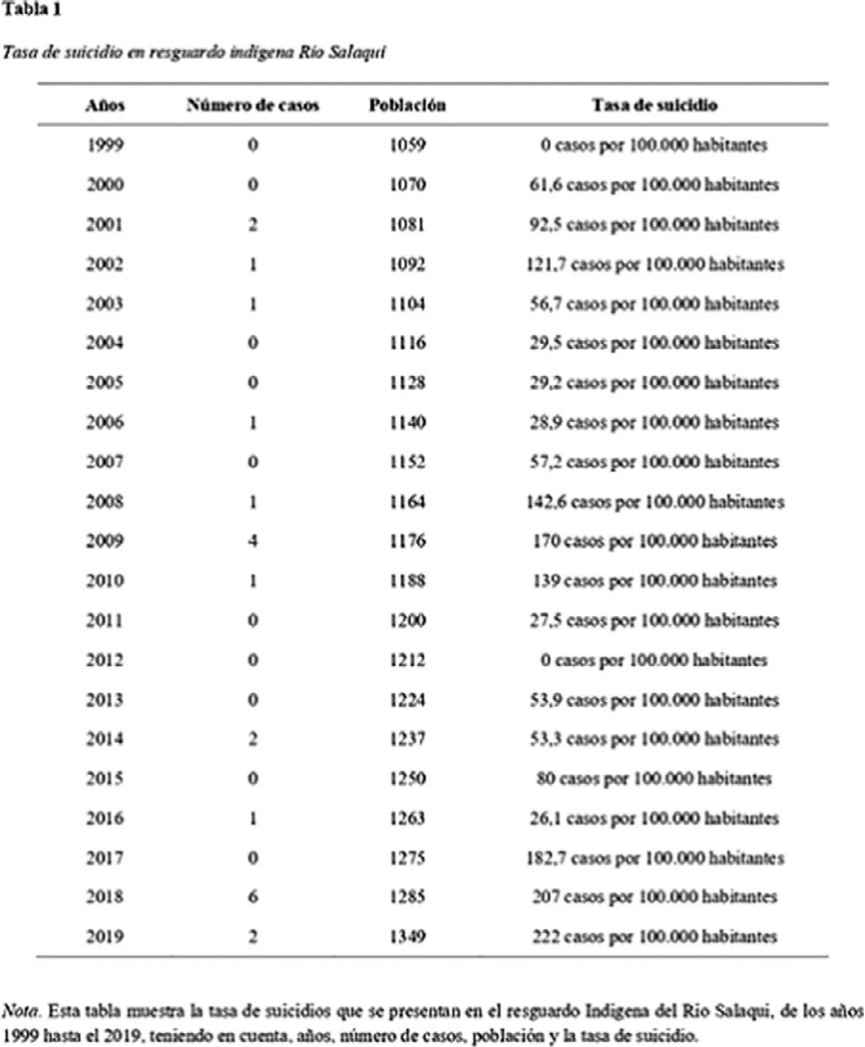

**Image 2:**

**Figure:**
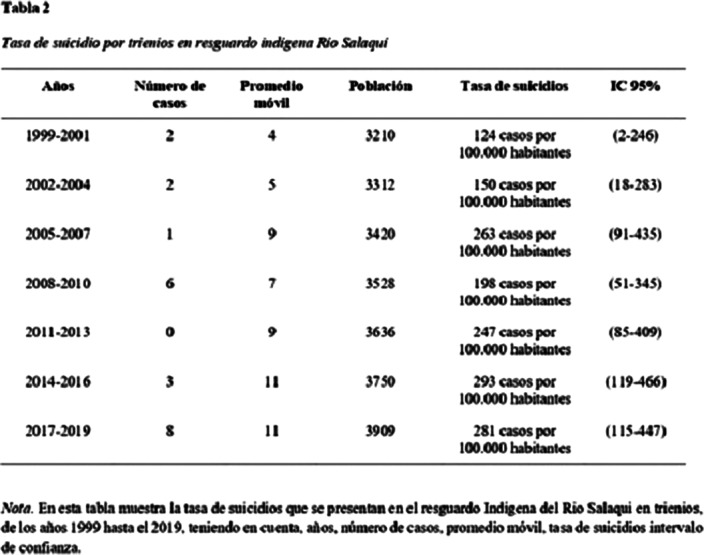

**Image 3:**

**Figure:**
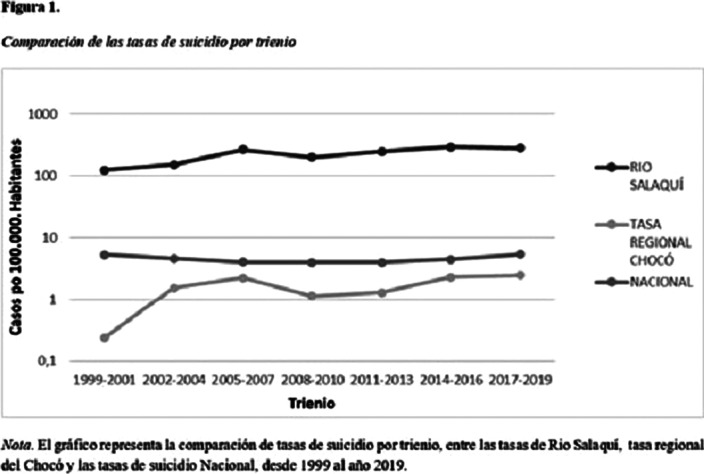

**Conclusions:**

During the development of the study is evident that in the indigenous reservation in the Salaqui river the suicide rate is significantly higher than in the national rates, it is even higher than the worldwide rate of suicide since the year 2001.

**Disclosure of Interest:**

None Declared

